# Identifying reproducible cancer-associated highly expressed genes with important functional significances using multiple datasets

**DOI:** 10.1038/srep36227

**Published:** 2016-10-31

**Authors:** Haiyan Huang, Xiangyu Li, You Guo, Yuncong Zhang, Xusheng Deng, Lufei Chen, Jiahui Zhang, Zheng Guo, Lu Ao

**Affiliations:** 1 Department of Bioinformatics, Key Laboratory of Ministry of Education for Gastrointestinal Cancer, Fujian Medical University, Fuzhou, 350108; 2 Department of Preventive Medicine, School of Basic Medicine Sciences, Gannan Medical University, Ganzhou, 341000.

## Abstract

Identifying differentially expressed (DE) genes between cancer and normal tissues is of basic importance for studying cancer mechanisms. However, current methods, such as the commonly used Significance Analysis of Microarrays (SAM), are biased to genes with low expression levels. Recently, we proposed an algorithm, named the pairwise difference (PD) algorithm, to identify highly expressed DE genes based on reproducibility evaluation of top-ranked expression differences between paired technical replicates of cells under two experimental conditions. In this study, we extended the application of the algorithm to the identification of DE genes between two types of tissue samples (biological replicates) based on several independent datasets or sub-datasets of a dataset, by constructing multiple paired average gene expression profiles for the two types of samples. Using multiple datasets for lung and esophageal cancers, we demonstrated that PD could identify many DE genes highly expressed in both cancer and normal tissues that tended to be missed by the commonly used SAM. These highly expressed DE genes, including many housekeeping genes, were significantly enriched in many conservative pathways, such as ribosome, proteasome, phagosome and TNF signaling pathways with important functional significances in oncogenesis.

The high-throughput gene expression profiling technologies facilitate screening expression levels for thousands of genes simultaneously. One of the main objectives for analyzing gene expression profiles is to identify genes differentially expressed (DE) in cancer compared with normal control^1^. Many methods have been proposed to identify DE genes^2^,^3^,^4^,^5^ and a popular choice is Significance Analysis of Microarrays (SAM) based on *t*-test statistic^6^. It has been reported that the *t*-test is biased to genes with low expression levels^3^,^6^ because a gene with low expression level may have a large absolute *t*-statistic due to its small variance, even when its mean difference between two conditions is small^7^. SAM was proposed to correct this bias. However, due to logarithmic transformation of data in SAM, the differences of log-scaled expression levels between two conditions are actually the logarithms of their fold change (FC) ratios. Because genes with low expression levels are more likely to reach large FCs than genes with high expression levels, SAM is also biased to genes with low expression levels^8^. Compared with genes expressed at low levels, genes expressed at high levels are more likely to be involved in some functionally conserved pathways such as oxidative phosphorylation^9^, glutathione metabolism^10^,^11^,^12^ and proteasome^13^ with important biological significances.

In a recent study^8^, we have proposed an algorithm, named the pairwise difference (PD) algorithm, to identify DE genes in small-scale cell line experiments, which typically measure only two or three technical replicates for each of two different experimental conditions, respectively. Briefly, by pairing technical replicates under two conditions, the algorithm identifies DE genes with top-ranked absolute expression differences between the two types of cells which are significantly reproducible in independent paired technical replicates^8^. Compared with SAM and other commonly used methods, PD can exclusively identify many DE genes with high expression levels in both two types of cells^8^. However, this algorithm cannot be used directly to identify DE genes between two types of tissue samples (e.g., cancer and normal control) because the biological replicates of each type of tissue may have large between-individual differences.

In this study, in consideration that tissue samples are biological replicates with between-individual differences, we averaged the gene expression profiles separately for two types of samples in a dataset to construct a cancer-normal pair, and then applied PD to identify DE genes using multiple cancer-normal pairs separately constructed from several independent datasets or sub-datasets of a dataset. Using datasets for lung cancer and esophagus cancer, we demonstrated the applicability and power of this strategy in finding functionally important DE genes highly expressed in both cancer and normal tissues that tend to be missed by SAM.

## Results

### The applicability of the PD algorithm to multiple datasets

**Figure 1:**
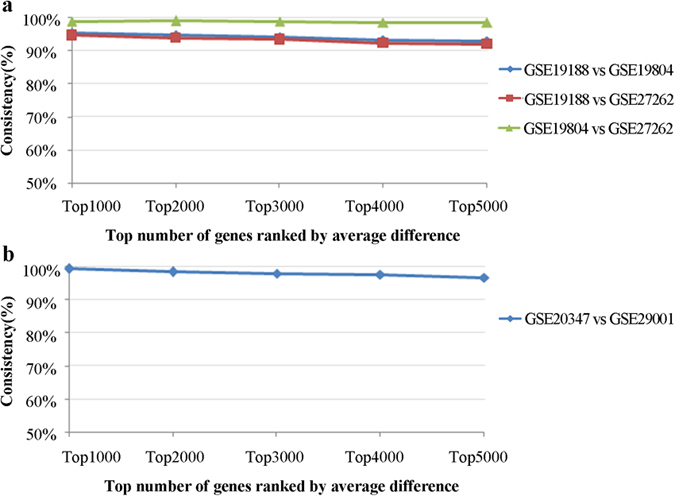
Consistency scores between two datasets for a cancer. The consistency scores between the top *n (n* = 1000, 2000, 3000, 4000, 5000) genes ranked by absolute average expression differences for every two cancer-normal pairs were evaluated in (**a**) three datasets for lung cancer (GSE19188, GSE19804 and GSE27262). and (**b**) two datasets for esophagus cancer (GSE20347 and GSE29001).

Firstly, for each of the three datasets for lung cancer and normal samples (see Table 1), we separately averaged the gene expression profiles for cancer and normal samples in each datasets to construct a paired average gene expression profiles, referred to as a cancer-normal pair. Then, for every cancer-normal pair, all genes were ranked according to their absolute average differences (AD) of expression levels between cancer and normal samples in descending order. As shown in Fig. 1a, the consistency scores of the deregulation directions of the top *n (n* = 1000, 2000, 3000, 4000, 5000) genes between every two cancer-normal pairs were all higher than 91.8%, which were all significantly higher than what expected by chance (binomial test, all *p* < 2.2 × 10^−16^) (see *Methods* for details). We did similar analyses in two datasets for esophagus cancer (Table 1) and found that the consistency scores of the deregulation directions of the top *n (n* = 1000, 2000, 3000, 4000, 5000) genes between the two datasets were all higher than 96.42%, as shown in Fig. 1b. These results suggested that the differential expression signals between every two independent cancer-normal pairs for a particular cancer were significantly reproducible.

We further did a random experiment to show that the differential expression signals were irreproducible when there were no phenotype differences between two groups of samples. Using the GSE19804 dataset with 60 lung cancer samples and 60 normal samples, we randomly permuted sample labels two times to produce two datasets of artificial “cancer” and “normal” samples, and then calculated the consistency score of the deregulation directions of the top 1000 genes sorted by the average expressions difference between the two artificial cancer-normal pairs. The random experiment was repeated 1000 times. As expected, the average of the 1000 consistency scores was 49.83% with 0.1954 of standard deviation. These results suggested that the differential expression signals were irreproducible when there were no phenotype differences between two groups of samples.

Then, regarding every cancer-normal pair as an independent pair of technical replicates, we used the PD algorithm to identify reproducible DE genes between the lung cancer and normal control of three datasets. The two parameters of the algorithm, the initial step and the consistency threshold, were set as 300 and 95%, respectively, as suggested previously^8^. With the above two parameters, PD identified a list of 6,092 DE genes for lung cancer, and this list of DE genes was denoted as C3. In comparison, 10,865, 12,287 and 10,945 DE genes were identified by SAM with 5% FDR control in the GSE19188, GSE19804 and GSE27262 datasets, respectively. The consistency scores of the overlapped DE genes between C3 and the DE genes identified by SAM in the three datasets were 99.83%, 100% and 100%, respectively (Table 2). Similarly, PD identified 3,498 DE genes based on the two datasets of esophagus cancer, denoted as C2, and the consistency scores with DE genes identified by SAM in the two datasets were both 100% (Table 2).

**Figure 2:**
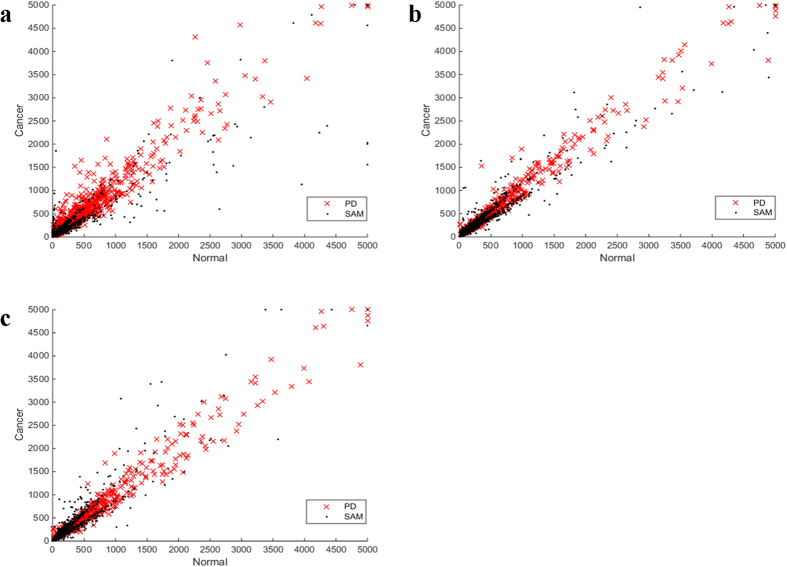
The distributions of the average expression levels for DE genes identified exclusively by PD or SAM for lung cancer. Red crosses represent the DE genes exclusively identified by PD in C3, and black dots represent the DE genes exclusively identified by SAM in datasets (**a**) GSE19188, (**b**) GSE19804, (**c**) GSE27262, respectively. The average expression levels of DE genes in normal samples (x-axis) and cancer samples (y-axis) were plotted. The average expression levels above 5,000 were set to 5,000.

On the other hand, approximately 9.3–22.1% of the DE genes in C3 identified by PD were not identified by SAM. As shown in Fig. 2, the average expression levels of the DE genes exclusively identified by PD were rather high in both cancer and normal samples of the three datasets, while the average expression levels of most DE genes exclusively identified by SAM were quite low in cancer and/or normal samples. Similar results were observed based on the two datasets for esophagus cancer (Supplementary Figure S1). Thus, the PD algorithm can identify DE genes expressed highly in both cancer and normal tissues, which tend to be missed by SAM.

### The applicability of the PD algorithm to a single dataset

We used the dataset GSE27262 for 25 lung cancer samples and 25 normal samples to exemplify the feasibility of the PD algorithm in analyzing a single dataset. Firstly, we divided this dataset evenly into two sub-datasets according to the GSM series numbers of samples: set 1 and set 2 with 12 and 13 pairs of cancer and normal samples, respectively (Table 3). Then, we transformed the two sub-datasets into two independent cancer-normal pairs of averaged gene expression profiles. With the same parameter setting as above, PD identified 3,789 DE genes, denoted as S2. 3,386 of these 3,789 DE genes overlapped with C3 and the consistency score between S2 and C3 was 100%. When dividing the GSE27262 into four small sub-datasets (Table 3), PD identified 4,157 DE genes, denoted as S4. The consistency score between S4 and C3 was 99.94%.

Similarly, when dividing the dataset GSE29001 evenly into two and four small sub-datasets, respectively, PD identified 1,738 and 2,298 DE genes for esophagus cancer. The consistency scores between the two lists of DE genes with the DE genes in C2 were 100% and 99.88% (Table 4), respectively.

Taking together, the above results demonstrated that PD can work well by dividing a dataset evenly into several sub-datasets with sample sizes as small as about six for each type of samples.

### Significant functional pathways detected by the PD algorithm

Here, we used the above dataset GSE27262 for lung cancer and the dataset GSE29001 for esophagus cancer to demonstrate that most of the pathways significantly enriched with DE genes found by PD tend to be missed by SAM.

**Figure 3:**
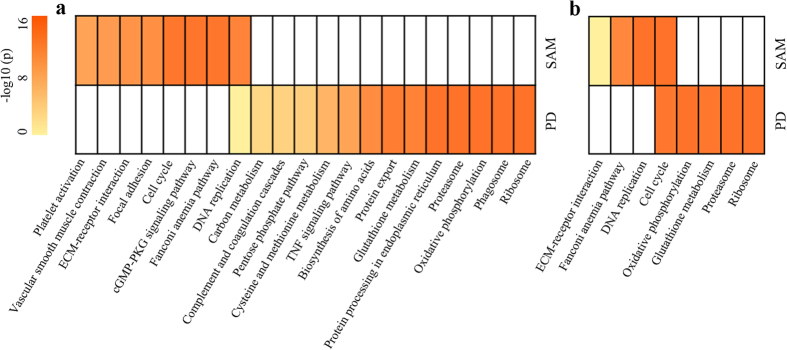
The comparison of functional pathways enriched with DE genes separately identified by PD and SAM. The biological pathways significantly enriched with DE genes identified by PD (using two subsets of each dataset, S2) and by SAM in (**a**) GSE27262 for lung cancer, (**b**) GSE29001 for esophagus cancer. The most significant DE genes identified by SAM, with the same number of the DE genes found by PD, were used for pathway enrichment analyses. The *p* values of the KEGG pathways were adjusted by Benjamini and Hochberg (FDR = 10%), and −log10(*p*) was used to generate the heat map.

With 10% FDR control, the DE genes in S2 found by PD for lung cancer were significantly enriched in 14 pathways (Fig. 3a). However, none of these pathways was identified as significant by enrichment analysis with the same FDR control for the 10,945 DE genes found by SAM with 5% FDR control. When focusing on the most significant DE genes found by SAM, with the same number of DE genes in S2, 13 of the 14 significant pathways were still unfound (Fig. 3a). Besides, the DNA replication pathway^19^ commonly found by PD and SAM, the other 13 significant pathways are mainly associated with lung cancer, including pentose phosphate pathway^20^, oxidative phosphorylation^9^,^21^, cysteine and methionine metabolism^22^, glutathione metabolism^10^,^11^,^12^, biosynthesis of amino acids^23^, ribosome^24^, proteasome^13^, protein processing in endoplasmic reticulum^25^,^26^, phagosome^27^ and TNF signaling pathway^28^. These conservative pathways included many DE genes highly expressed in both cancer and normal tissues, which tended to be missed by SAM. For example, among the 16 DE genes found exclusively by PD in the TNF signaling pathway, the average expression level of CCL2 was ranked at the top 3.2% and 1% of all the measured genes in the cancer and normal samples, respectively. The difference between the average expression level of this gene in the cancer samples and its average expression level in the normal samples was as large as 1678.72, whereas the average of the corresponding differences for all the DE genes identified by SAM was only 245.03. It has been reported that this gene may play an important role in the development of lung cancer^29^. For another example, the average expression level of TNFAIP3 was ranked at the top 7.4% and 3.2% of all the measured genes in the cancer and normal samples, respectively. The difference between the average expression level of this gene in the cancer samples and its average expression level in the normal samples was 625.43. This gene has been reported as a negative regulator of NF-kappa B activation as well as TNF-mediated apoptosis^30^ and its underexpression can promote the progression of lung cancer^31^. The detailed information about these 16 DE genes was shown in Supplementary Table S1.

Similarly for esophagus cancer, the four pathways significantly enriched with DE genes in S2 identified by PD were all missed by SAM (Fig. 3b). These significant pathways included pathways for oxidative phosphorylation^32^, glutathione metabolism^33^, ribosome^34^,^35^ and proteasome^36^.

The above pathway enrichment analyses demonstrated that the PD algorithm can capture important cancer-associated pathways with highly expressed DE genes, including many housekeeping genes (see *Discussion*), which might play important roles in oncogenesis, whereas most of these pathways tend to be missed by SAM. The results also provided extra evidence supporting the reliability of the DE genes found by PD because a list of DE genes can be significantly enriched in pathways only when it contains sufficient real DE genes^37^,^38^.

## Discussion

In this paper, we extended the application of the PD algorithm to the identification of DE genes between cancer and normal tissue samples based on several independent datasets or sub-datasets of a dataset. The application results for lung and esophageal cancer showed that PD can exclusively identify many DE genes with high expression levels in both cancer and normal samples, which tend to be missed by the commonly used SAM. Functional enrichment analyses of DE genes identified by PD showed that it can exclusively identify many significant biological pathways related to the development of cancers. Especially, the results demonstrated that the PD algorithm could efficiently identify DE genes by dividing a dataset evenly into several sub-datasets with sample sizes as small as about six for each type of samples. In general, for researchers with their own experimental data, we would recommend them making use of independent datasets in public data sources, in cases that such data exist, in order to increase the power and accuracy of biological discovery.

Notably, in our functional analysis examples for lung cancer and esophagus cancer, four pathways were commonly identified by PD but missed by SAM. These four pathways were well known cancer-related pathways for oxidative phosphorylation, glutathione metabolism, ribosome and proteasome. These biological pathways are related to two important cancer hallmarks, the metabolic network (the oxidative phosphorylation and glutathione metabolism pathways) and genome duplication network (ribosome) according to the cancer hallmarks network framework proposed by Wang *et al*.^39^. Reprogramming of metabolism is an important mechanism supporting the growth and division of cancer cell^40^. Genome duplication plays an important role on tumor formation and can activate several cancer hallmarks network^41^,^42^. These conservative cancer hallmarks or pathways all included many highly expressed housekeeping genes playing essential roles in the pathogenesis of cancer. For example, in the ribosome pathway, among the 45 DE genes found exclusively by PD in the GSE27262 dataset for lung cancer (Supplementary Table S2), 35 genes were housekeeping genes reported by Zhu *et al*.^43^. The average expression levels of these 35 housekeeping genes were all ranked among the top 20% of all the measured genes in both the cancer and normal samples. It is known that housekeeping genes maintain the basic needs for a cell to survive^44^,^45^,^46^, and thus their deregulations tend to induce human diseases including cancer^47^,^48^. For examples, the overexpression of RPSA may be positively correlated with the angiogenesis of lung cancer^49^,^50^, the overexpression of RPL19 promotes malignant proliferation of lung cancer cells^51^, and the underexpression of RPS3, a critical regulator of DNA repair and apoptosis^52^, might accelerate the development of lung cancer. Such cancer-related housekeeping genes tend to be evolutionarily conserved and play critical roles in carcinogenesis together with tissue-specific less-conservative cancer-related genes^53^.

Although PD can exclusively identify many important cancer-associated genes with high expression levels which play important functional roles in carcinogenesis, it has its own shortcomings. A major limitation is that it still cannot obtain DE genes with FDR control. Obviously, the higher the consistency threshold was set, the lower the rate of false positives of DE genes identified between two independent sample pairs. However, the FDR has a complex relationship with the parameter of consistency threshold. Besides, some DE genes and pathways identified by SAM were missed by PD which is biased to genes with high expressions. For example, DE genes identified by SAM from the dataset GSE27262 for lung cancer were enriched in the fanconi anemia pathway related with risk of lung adenocarcinoma^54^,^55^. However, this pathway was missed by DE genes identified by PD. In this pathway, 13 DE genes were identified by SAM but not by PD. The average expression levels of the 13 genes were among the bottom 70% and 61% of all the measured genes of all the cancer and normal samples, respectively. These results demonstrate that, different from SAM, PD tends to miss DE genes with low expression levels. Therefore, the PD algorithm is not a substitution but an effective complement to current approaches for analyzing DE genes of tissue datasets with biological replicates.

## Methods

### Data and data pre-processing

Multiple gene expression datasets for lung cancer and esophageal cancer were collected from Gene Expression Omnibus (GEO)^56^. Detail information about these datasets used in this study were described in Table 1. For each dataset, the raw data (.CEL files) was pre-processed using the robust average (RMA) algorithm^57^,^58^. Then each probe-set ID was matched to its Entrez gene ID. If multiple probesets were matched to the same gene, the expression value for the gene was referred to as the arithmetic mean of the values of the multiple probesets (on the log2 scale).

### Identification of reproducible DE genes

The pairwise difference (PD) algorithm^8^ was originally designed for analyzing small-scale cell line data with two or three technical replicates for each of two different cell lines. Since technical replicates for a cell line have no biological difference, every two independent pairs of technical replicates for two different cell lines can be regarded as independent experiments to identify DE genes through reproducibility evaluation. However, because tissue samples from different individuals are biological replicates with large biological variations among individuals, every two paired samples for two types of tissues cannot be regarded as reproducible independent experiments. In order to reduce the influence of biological variations among samples with the same phenotype, we used several independent datasets to construct multiple cancer-normal pairs by averaging a set of gene expression profiles separately for each of the two phenotypes. Specifically, for each dataset, we calculated the mean non-log-transformed expression values of each gene in the normal samples (type N) and cancer samples (type C), respectively, to form a paired average gene expression profiles for cancer and normal tissues. For a given pair *j* consisting of one type N sample and one type C sample, the mean values of gene *i* in the type N sample and type C sample, denoted as 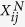 and 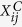, respectively, were calculated as following:


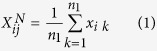



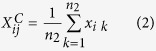


where *n*_1_ and *n*_2_ were the numbers of samples in type N and type C, respectively. *x*_*ik*_ was the expression value of gene *i* in a type N or type C sample.

Then, for gene *i*, the average expression difference between two phenotypes of a given cancer-normal pair *j*, denoted as *D*_*ij*_, was calculated as following:


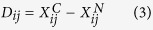


If the value was larger (or smaller) than zero, then gene *i* was defined as up-regulation (or down-regulation) in type C sample. Regarding multiple cancer-normal pairs constructed from independent datasets as independent experiments, we could identify DE genes through reproducibility evaluation with the same PD algorithm descried in details in our original paper^8^. Briefly, all genes in each cancer-normal pair were sorted in descending order by their absolute pairwise expression differences between two phenotypes and divided into blocks by the initial step of 300. The significantly reproducible DE gene lists between the decreasingly ranked blocks of each two independent pairs were identified if their consistency scores were higher than a pre-settled consistency threshold (here, 95%).

### Reproducibility evaluation of two DE gene lists

For two DE gene lists from two different datasets sharing *k* DE genes, of which *s* genes had the consistent directions (either up-regulation or down-regulation) in type C samples, the consistency score was calculated as *s/k*. The cumulative binomial distribution model^59^ was used to estimate the probability of observing at least *s* of *k* DE genes with the consistent directions by chance:


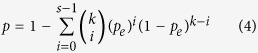


in which *p*_e_ is the probability of one gene having the consistent direction in two DE gene lists by random chance (here, *p*_e_ = 0.5). A DE genes list is considered significantly reproducible if the *p* value of the consistency score is <0.01.

### Pathway enrichment analysis

Functional enrichment analysis was done based on the Kyoto Encyclopaedia of Genes and Genomes^60^. The hypergeometric distribution model was used to identify biological pathways that were significantly enriched with DE genes^61^, the probability of observing at least *k* genes in a pathway by chance can be computed as follow:


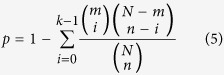


*n* is the number of DE genes identified from *N* genes in a dataset and *k* of them are annotated in a pathway with *m* genes.

The *p* values were adjusted using the Benjamini and Hochberg procedure^62^, controlling the False Discovery Rate (FDR) at the 10% level.

## Additional Information

**How to cite this article**: Huang, H. *et al*. Identifying reproducible cancer-associated highly expressed genes with important functional significances using multiple datasets. *Sci. Rep.* **6**, 36227; doi: 10.1038/srep36227 (2016).

**Publisher’s note**: Springer Nature remains neutral with regard to jurisdictional claims in published maps and institutional affiliations.

## Supplementary Material

Supplementary Information

## Figures and Tables

**Table 1:** Description of the datasets used in this study.

Tissue	GEO accession	Platform	Cancer	Normal	Reference
Lung	GSE19188	GPL570	91	65	Hou, J. *et al*.^14^
	GSE19804		60	60	Lu, T.P. *et al*.^15^
	GSE27262		25	25	Wei, T.Y. *et al*.^16^
Esophagus	GSE29001	GPL571	21	24	Yan, W. *et al*.^17^
	GSE20347		17	17	Hu, N. *et al*.^18^

**Table 2:** The consistency scores of the DE genes identified by both PD and SAM.

Tissue	Dataset	PD	SAM	Overlap	Consistency	Consistency score	*P*
Lung	GSE19188	6092	10865	4744	4736	99.83%	<2.2 × 10^−16^
	GSE19804		12287	5488	5488	100.00%	<2.2 × 10^−16^
	GSE27262		10945	5524	5524	100.00%	<2.2 × 10^−16^
Esophagus	GSE20347	3498	6311	3057	3057	100.00%	<2.2 × 10^−16^
	GSE29001		5882	2785	2785	100.00%	<2.2 × 10^−16^

Note: Overlap, the number of the DE genes identified by both PD and SAM; Consistency, the number of the DE genes with the same deregulation directions (either up-regulation or down-regulation); *P*, the significance level of reproducibility.

**Table 3:** The consistency scores of DE genes identified by PD from sub-datasets of GSE27262 and three datasets for lung cancer.

List	Sub-dataset	Cancer	Normal	DE genes	Overlap	Consistency score	*P*
S2	set 1	12	12	3789	3386	100.00%	<2.2 × 10^−16^
	set 2	13	13				
S4	set 1	6	6	4157	3119	99.94%	<2.2×10^−16^
	set 2	6	6				
	set 3	6	6				
	set 4	7	7				

Note: S2 and S4 separately represent two (set 1 and set 2) and four (set 1, set 2, set 3 and set 4) small sub-datasets with cancer and normal samples by evenly dividing GSE27262 according to the GSM series numbers of samples. Cancer/Normal, the number of cancer/normal samples in each sub-dataset; DE genes, the number of genes identified by PD in S2 and S4; Overlap, the number of the DE genes shared by S2 (or S4) and C3; *P*, the significance level of reproducibility.

**Table 4:** The consistency scores of DE genes identified by PD from sub-datasets of GSE29001 and two datasets for esophagus cancer.

List	Sub-datasets	Cancer	Normal	DE genes	Overlaps	Consistency score	*P*
S2	Set 1	10	12	1738	1651	100.00%	<2.2 × 10–16
	Set 2	11	12				
S4	Set 1	5	6	2298	1724	99.88%	<2.2 × 10–16
	Set 2	5	6				
	Set 3	5	6				
	Set 4	6	6				

Note: See Note for Table 3.

## References

[b1] Feten G., Aastveit A. H., Snipen L. & Almoy T. A discussion concerning the inclusion of variety effect when analysis of variance is used to detect differentially expressed genes. Gene Regul Syst Bio 1, 43–47 (2007). PMC2759136 19936077

[b2] Breitling R., Armengaud P., Amtmann A. & Herzyk P. Rank products: a simple, yet powerful, new method to detect differentially regulated genes in replicated microarray experiments. FEBS Lett 573, 83–92, doi: 10.1016/j.febslet.2004.07.055 (2004). 15327980

[b3] Smyth G. K. Linear models and empirical bayes methods for assessing differential expression in microarray experiments. Stat Appl Genet Mol Biol 3, Article 3, doi: 10.2202/1544-6115.1027 (2004). 16646809

[b4] Pounds S. & Rai S. N. Assumption Adequacy Averaging as a Concept to Develop More Robust Methods for Differential Gene Expression Analysis. Comput Stat Data Anal 53, 1604–1612, doi: 10.1016/j.csda.2008.05.010 (2009). 20161327 PMC2678745

[b5] McCarthy D. J. & Smyth G. K. Testing significance relative to a fold-change threshold is a TREAT. Bioinformatics 25, 765–771, doi: 10.1093/bioinformatics/btp053 (2009). 19176553 PMC2654802

[b6] Tusher V. G., Tibshirani R. & Chu G. Significance analysis of microarrays applied to the ionizing radiation response. Proc Natl Acad Sci USA 98, 5116–5121, doi: 10.1073/pnas.091062498 (2001). 11309499 PMC33173

[b7] Yang D., Parrish R. S. & Brock G. N. Empirical evaluation of consistency and accuracy of methods to detect differentially expressed genes based on microarray data. Comput Biol Med 46, 1–10, doi: 10.1016/j.compbiomed.2013.12.002 (2014). 24529200 PMC3993975

[b8] Ao L. . Identification of reproducible drug-resistance-related dysregulated genes in small-scale cancer cell line experiments. Sci Rep 5, 11895, doi: 10.1038/srep11895 (2015). 26173481 PMC4502408

[b9] De Rosa V. . Reversal of Warburg Effect and Reactivation of Oxidative Phosphorylation by Differential Inhibition of EGFR Signaling Pathways in Non-Small Cell Lung Cancer. Clin Cancer Res 21, 5110–5120, doi: 10.1158/1078-0432.CCR-15-0375 (2015). 26216352

[b10] Sun Z. . Genetic variation in glutathione metabolism and DNA repair genes predicts survival of small-cell lung cancer patients. Ann Oncol 21, 2011–2016, doi: 10.1093/annonc/mdq212 (2010). 20439344 PMC2946864

[b11] Yang P., Ebbert J. O., Sun Z. & Weinshilboum R. M. Role of the glutathione metabolic pathway in lung cancer treatment and prognosis: a review. J Clin Oncol 24, 1761–1769, doi: 10.1200/JCO.2005.02.7110 (2006). 16603718

[b12] Blair S. L. . Glutathione metabolism in patients with non-small cell lung cancers. Cancer Res 57, 152–155 (1997). 8988057

[b13] Jagoe R. T., Redfern C. P., Roberts R. G., Gibson G. J. & Goodship T. H. Skeletal muscle mRNA levels for cathepsin B, but not components of the ubiquitin-proteasome pathway, are increased in patients with lung cancer referred for thoracotomy. Clin Sci (Lond) 102, 353–361 (2002). 11869177

[b14] Hou J. . Gene expression-based classification of non-small cell lung carcinomas and survival prediction. PLoS One 5, e10312, doi: 10.1371/journal.pone.0010312 (2010). 20421987 PMC2858668

[b15] Lu T. P. . Identification of a novel biomarker, SEMA5A, for non-small cell lung carcinoma in nonsmoking women. Cancer Epidemiol Biomarkers Prev 19, 2590–2597, doi: 10.1158/1055-9965.EPI-10-0332 (2010). 20802022

[b16] Wei T. Y. . Methylosome protein 50 promotes androgen- and estrogen-independent tumorigenesis. Cell Signal 26, 2940–2950, doi: 10.1016/j.cellsig.2014.09.014 (2014). 25277535

[b17] Yan W. . Identification of unique expression signatures and therapeutic targets in esophageal squamous cell carcinoma. BMC Res Notes 5, 73, doi: 10.1186/1756-0500-5-73 (2012). PMC3283499 22280838

[b18] Hu N. . Genome wide analysis of DNA copy number neutral loss of heterozygosity (CNNLOH) and its relation to gene expression in esophageal squamous cell carcinoma. BMC Genomics, 11, 576, doi: 10.1186/1471-2164-11-576 (2010). PMC3091724 20955586

[b19] Macheret M. & Halazonetis T. D. DNA replication stress as a hallmark of cancer. Annu Rev Pathol 10, 425–448, doi: 10.1146/annurev-pathol-012414-040424 (2015). 25621662

[b20] Rao X. . O-GlcNAcylation of G6PD promotes the pentose phosphate pathway and tumor growth. Nat Commun 6, 8468, doi: 10.1038/ncomms9468 (2015). 26399441 PMC4598839

[b21] Gao C., Shen Y., Jin F., Miao Y. & Qiu X. Cancer Stem Cells in Small Cell Lung Cancer Cell Line H446: Higher Dependency on Oxidative Phosphorylation and Mitochondrial Substrate-Level Phosphorylation than Non-Stem Cancer Cells. PLoS One 11, e0154576, doi: 10.1371/journal.pone.0154576 (2016). 27167619 PMC4863974

[b22] Shi Q. . Polymorphisms of methionine synthase and methionine synthase reductase and risk of lung cancer: a case-control analysis. Pharmacogenet Genomics 15, 547–555 (2005). 16006998 10.1097/01.fpc.0000170916.96650.70

[b23] Imai H. . Inhibition of L-type amino acid transporter 1 has antitumor activity in non-small cell lung cancer. Anticancer Res 30, 4819–4828 (2010). 21187458

[b24] Tsai H. Y. . Endoplasmic reticulum ribosome-binding protein 1 (RRBP1) overexpression is frequently found in lung cancer patients and alleviates intracellular stress-induced apoptosis through the enhancement of GRP78. Oncogene 32, 4921–4931, doi: 10.1038/onc.2012.514 (2013). 23318453

[b25] Hung J. Y. . Oxidative and endoplasmic reticulum stress signaling are involved in dehydrocostuslactone-mediated apoptosis in human non-small cell lung cancer cells. Lung Cancer 68, 355–365, doi: 10.1016/j.lungcan.2009.07.017 (2010). 19700217

[b26] Kang K. A. . Fisetin induces apoptosis and endoplasmic reticulum stress in human non-small cell lung cancer through inhibition of the MAPK signaling pathway. Tumour Biol, doi: 10.1007/s13277-016-4864-x (2016). 26797785

[b27] Scagliotti G. Proteasome inhibitors in lung cancer. Crit Rev Oncol Hematol 58, 177–189, doi: 10.1016/j.critrevonc.2005.12.001 (2006). 16427303

[b28] Chu W. M. Tumor necrosis factor. Cancer Lett 328, 222–225, doi: 10.1016/j.canlet.2012.10.014 (2013). 23085193 PMC3732748

[b29] Li X., Lin F. & Zhou H. Genetic polymorphism rs3760396 of the chemokine (C-C motif) ligand 2 gene (CCL2) associated with the susceptibility of lung cancer in a pathological subtype-specific manner in Han-ancestry Chinese: a case control study. BMC Cancer 16, 298, doi: 10.1186/s12885-016-2328-8 (2016). 27145753 PMC4855406

[b30] Langsch S. . miR-29b Mediates NF-kappaB Signaling in KRAS-Induced Non-Small Cell Lung Cancers. Cancer Res, doi: 10.1158/0008-5472.CAN-15-2580 (2016). 27199349

[b31] Yamamoto K., Ito S., Hanafusa H., Shimizu K. & Ouchida M. Uncovering Direct Targets of MiR-19a Involved in Lung Cancer Progression. PLoS One 10, e0137887, doi: 10.1371/journal.pone.0137887 (2015). 26367773 PMC4569347

[b32] Suchorolski M. T., Paulson T. G., Sanchez C. A., Hockenbery D. & Reid B. J. Warburg and Crabtree effects in premalignant Barrett’s esophagus cell lines with active mitochondria. PLoS One 8, e56884, doi: 10.1371/journal.pone.0056884 (2013). 23460817 PMC3584058

[b33] Zhang Y. H., Lin J. X. & Vilcek J. Interleukin-6 induction by tumor necrosis factor and interleukin-1 in human fibroblasts involves activation of a nuclear factor binding to a kappa B-like sequence. Mol Cell Biol 10, 3818–3823 (1990). 2192263 10.1128/mcb.10.7.3818-3823.1990 PMC360846

[b34] Kim S. H., Jang Y. H., Chau G. C., Pyo S. & Um S. H. Prognostic significance and function of phosphorylated ribosomal protein S6 in esophageal squamous cell carcinoma. Mod Pathol 26, 327–335, doi: 10.1038/modpathol.2012.161 (2013). 22996377

[b35] Wang X. . Down-regulation of 5S rRNA by miR-150 and miR-383 enhances c-Myc-rpL11 interaction and inhibits proliferation of esophageal squamous carcinoma cells. FEBS Lett 589, 3989–3997, doi: 10.1016/j.febslet.2015.11.012 (2015). 26606907

[b36] Zhang W. G. . Inhibitory effect of ubiquitin-proteasome pathway on proliferation of esophageal carcinoma cells. World J Gastroenterol 10, 2779–2784 (2004). 15334669 10.3748/wjg.v10.i19.2779 PMC4572101

[b37] Yang D. . Gaining confidence in biological interpretation of the microarray data: the functional consistence of the significant GO categories. Bioinformatics 24, 265–271, doi: 10.1093/bioinformatics/btm558 (2008). 18006543

[b38] Zou J. . Revealing weak differential gene expressions and their reproducible functions associated with breast cancer metastasis. Comput Biol Chem 39, 1–5, doi: 10.1016/j.compbiolchem.2012.04.002 (2012). 22634492

[b39] Wang E. . Predictive genomics: a cancer hallmark network framework for predicting tumor clinical phenotypes using genome sequencing data. Seminars in cancer biology 30, 4–12, doi: 10.1016/j.semcancer.2014.04.002 (2015). 24747696

[b40] Hanahan D. & Weinberg R. A. Hallmarks of cancer: the next generation. Cell 144, 646–674, doi: 10.1016/j.cell.2011.02.013 (2011). 21376230

[b41] Wang E. . Cancer systems biology in the genome sequencing era: part 1, dissecting and modeling of tumor clones and their networks. Seminars in cancer biology 23, 279–285, doi: 10.1016/j.semcancer.2013.06.002 (2013). 23791722

[b42] Wang E. . Cancer systems biology in the genome sequencing era: part 2, evolutionary dynamics of tumor clonal networks and drug resistance. Seminars in cancer biology 23, 286–292, doi: 10.1016/j.semcancer.2013.06.001 (2013). 23792107

[b43] Zhu J., He F., Song S., Wang J. & Yu J. How many human genes can be defined as housekeeping with current expression data? BMC Genomics 9, 172, doi: 10.1186/1471-2164-9-172 (2008). 18416810 PMC2396180

[b44] Yang L. . Comparative analysis of housekeeping and tissue-selective genes in human based on network topologies and biological properties. Mol Genet Genomics 291, 1227–1241, doi: 10.1007/s00438-016-1178-z (2016). 26897376

[b45] She X. . Definition, conservation and epigenetics of housekeeping and tissue-enriched genes. BMC Genomics 10, 269, doi: 10.1186/1471-2164-10-269 (2009). 19534766 PMC2706266

[b46] Shaw G. T., Shih E. S., Chen C. H. & Hwang M. J. Preservation of ranking order in the expression of human Housekeeping genes. PLoS One 6, e29314, doi: 10.1371/journal.pone.0029314 (2011). 22216246 PMC3245260

[b47] Rubie C. . Housekeeping gene variability in normal and cancerous colorectal, pancreatic, esophageal, gastric and hepatic tissues. Mol Cell Probes 19, 101–109, doi: 10.1016/j.mcp.2004.10.001 (2005). 15680211

[b48] Chen M. . Identification of human HK genes and gene expression regulation study in cancer from transcriptomics data analysis. PLoS One 8, e54082, doi: 10.1371/journal.pone.0054082 (2013). 23382867 PMC3561342

[b49] Fontanini G. . 67-Kilodalton laminin receptor expression correlates with worse prognostic indicators in non-small cell lung carcinomas. Clin Cancer Res 3, 227–231 (1997). 9815677

[b50] Moodley K. & Weiss S. F. Downregulation of the non-integrin laminin receptor reduces cellular viability by inducing apoptosis in lung and cervical cancer cells. PLoS One 8, e57409, doi: 10.1371/journal.pone.0057409 (2013). 23472084 PMC3589420

[b51] Kuroda K. . Identification of ribosomal protein L19 as a novel tumor antigen recognized by autologous cytotoxic T lymphocytes in lung adenocarcinoma. Cancer Sci 101, 46–53, doi: 10.1111/j.1349-7006.2009.01351.x (2010). 19799608 PMC11159900

[b52] Jang C. Y., Lee J. Y. & Kim J. RpS3, a DNA repair endonuclease and ribosomal protein, is involved in apoptosis. FEBS Lett 560, 81–85, doi: 10.1016/S0014-5793(04)00074-2 (2004). 14988002

[b53] Zhu J. . Viewing cancer genes from co-evolving gene modules. Bioinformatics 26, 919–924, doi: 10.1093/bioinformatics/btq055 (2010). 20176579

[b54] Yang S. Y. . Fanconi anemia genes in lung adenocarcinoma- a pathway-wide study on cancer susceptibility. J Biomed Sci 23, 23, doi: 10.1186/s12929-016-0240-9 (2016). 26842001 PMC4739091

[b55] Duan W. . Fanconi anemia repair pathway dysfunction, a potential therapeutic target in lung cancer. Front Oncol 4, 368, doi: 10.3389/fonc.2014.00368 (2014). 25566506 PMC4271581

[b56] Edgar R., Domrachev M. & Lash A. E. Gene Expression Omnibus: NCBI gene expression and hybridization array data repository. Nucleic Acids Res 30, 207–210 (2002). 11752295 10.1093/nar/30.1.207 PMC99122

[b57] Irizarry R. A. . Summaries of Affymetrix GeneChip probe level data. Nucleic Acids Res 31, e15 (2003). 12582260 10.1093/nar/gng015 PMC150247

[b58] Bolstad B. M., Irizarry R. A., Astrand M. & Speed T. P. A comparison of normalization methods for high density oligonucleotide array data based on variance and bias. Bioinformatics 19, 185–193 (2003). 12538238 10.1093/bioinformatics/19.2.185

[b59] Bahn A. K. Application of binomial distribution to medicine: comparison of one sample proportion to an expected proportion (for small samples). Evaluation of a new treatment. Evaluation of a risk factor. J Am Med Womens Assoc 24, 957–966 (1969). 4243043

[b60] Kanehisa M. & Goto S. KEGG: kyoto encyclopedia of genes and genomes. Nucleic Acids Res 28, 27–30 (2000). 10592173 10.1093/nar/28.1.27 PMC102409

[b61] Belanger B. F., Williams W. J. & Yin T. C. A flexible renewal process simulator for neural spike trains. IEEE Trans Biomed Eng 23, 262–266 (1976). 1262038 10.1109/tbme.1976.324641

[b62] Benjamini Y. H. Y. Controlling the False Discovery Rate: A Practical and Powerful Approach to Multiple Testing. J. Roy. Stat. Soc. 57, 289–300 (1995).

