# Supplementary material for: Identifying reproducible cancer-associated highly expressed genes with important functional significances using multiple datasets
Source: Sci Rep. 2016 Oct 31;6:36227. doi: 10.1038/srep36227 (PMC5086981; doi:10.1038/srep36227)
Supplement: Supplementary Information [file srep36227-s1.pdf]

Identifying reproducible cancer-associated highly expressed genes with important functional significances using multiple datasets

Haiyan Huang[1], Xiangyu Li[1], You Guo[1,2], Yuncong Zhang[1], Xusheng Deng[1], Lufei Chen[1], Jiahui Zhang , Zheng Guo[1*], Lu Ao[1*]

Contents

1 Supplementary Figures
2Supplementary Tables

1 Supplementary Figures

Supplementary Figure S1：The distributions of the average expression levels for DE genes identified exclusively by PD or SAM for esophagus cancer

Red crosses represent the DE genes exclusively identified by PD in C2, and black dots represent the DE genes exclusively identified by SAM in datasets a) GSE20347 and b) GSE29001, respectively. The average expression levels of DE genes in normal samples (x-axis) and cancer samples (y-axis) were plotted. The average expression levels above 5,000 were set to 5,000.

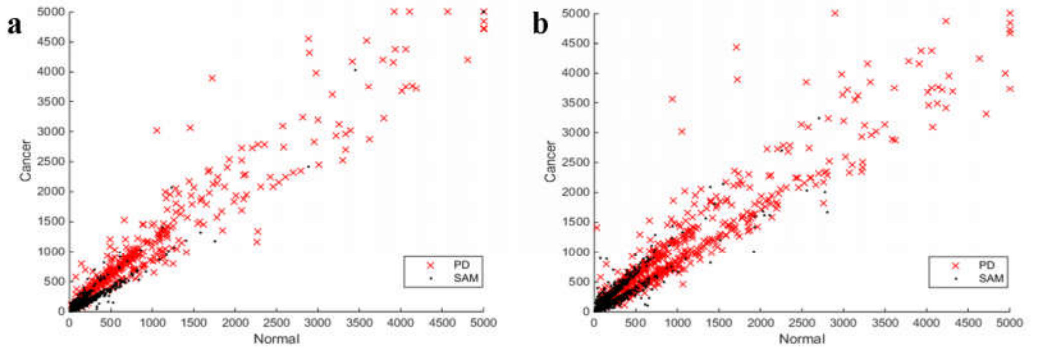

2 Supplementary Tables

Supplementary Table S1: The information of DE genes found by PD but missed by SAM in the TNF signaling pathway

Symbol	Rank in cancer[a]	Rank in normal[b]	deregulation directions	housekeeping genes
JAG1	5439	4213	0	N
AKT1	1014	1342	1	N
BIRC2	905	778	0	Y
FAS	4589	2639	0	N
BCL3	4079	2718	0	N
ICAM1	1084	636	0	Y
MAP3K5	1722	2142	1	N
MAPK3	1881	1436	0	N
MAPK9	2942	3833	1	N
CCL2	649	205	0	N
CCL20	2418	4292	1	N
TNFAIP3	1510	643	0	Y
IKBKG	3033	3790	1	Y
CFLAR	3741	2471	0	Y
CREE3	2112	2614	1	N
TAB2	2105	1676	0	Y

Note: All gene (20283 genes) were ranked according to their average expression levels in cancer samples and normal samples in descending order, respectively. [a] the rank of a gene in cancer samples, [b] the rank of a gene in normal samples. 0/1 represent the underexpression or overexpression of DE genes in cancer samples. Y/N represent that the gene is/isn't a housekeeping gene.

Supplementary Table S2: The information of DE genes found by PD but missed by SAM in the ribosome pathway

Symbol	Rank in cancer[a]	Rank in normal[b]	deregulation directions	housekeeping genes
RPSA	56	60	1	Y
RPL10A	581	687	1	Y
RPL6	54	58	1	Y
RPL8	50	57	1	Y
RPL12	49	59	1	Y
RPL15	958	773	0	Y
RPL19	32	44	1	Y
RPL24	30	35	1	Y
RPL27	25	29	1	Y
RPL30	31	37	1	Y
RPL29	63	73	1	Y
RPL31	971	853	0	Y
RPL32	16	16	0	Y
RPL37A	778	942	1	Y
RPL38	750	890	1	Y
RPL36A	43	66	1	Y
RPLP0	29	46	1	Y
RPS2	70	100	1	Y
RPS3	60	52	0	Y
RPS4X	15	17	1	Y
RPS5	38	55	1	Y
RPS7	61	68	1	Y
RPS8	85	104	1	Y
RPS10	24	31	1	Y
RPS13	19	25	1	Y
RPS19	440	557	1	Y
RPS20	21	21	0	Y
RPS21	428	629	1	Y
RPS23	296	246	0	Y
RPS24	490	738	1	Y
RPS26	121	143	1	N
RPS27	1099	898	0	Y
RPS29	22	27	1	Y
RPL35	66	74	1	Y
MRPL13	1764	2751	1	N
MRPL18	1063	1528	1	Y
MRPL15	1385	2140	1	Y
MRPL22	2206	2932	1	N

RSL24D1	1057	891	0	N
MRPL32	723	1125	1	N
MRPL11	2199	2631	1	N
MRPL1	2779	3286	1	N
MRPL10	2198	2655	1	N
RPL22L1	1804	3185	1	N
MRPL21	2971	3513	1	N

Note: See Note for Supplementary Table S2
